# Acute Physical Stress Preconditions the Heart Against Ischemia/Reperfusion Injury Through Activation of Sympathetic Nervous System

**DOI:** 10.5935/abc.20190189

**Published:** 2019-09

**Authors:** Alireza Imani, Hoda Parsa, Leila Gholami Chookalaei, Kamran Rakhshan, Masoomeh Golnazari, Mahdieh Faghihi

**Affiliations:** 1 Department of physiology - School of Medicine - Tehran University of Medical Sciences, Tehran - Iran; 2 Occupational Sleep Research Center - Baharloo Hospital - Tehran University of Medical Sciences, Tehran - Iran; 3 Department of physiology - School of Medicine - Iran University of Medical Sciences, Tehran - Iran; 4 Biology Department - Basic Sciences faculty - Hamedan Branch of Islamic Azad University, Hamedan - Iran

**Keywords:** Stress, Mechanical, Sympathetic Nervous System, Hypothalamo-Hypophyseal System, Ischemia, Sympathectomy

## Abstract

**Background:**

Stress is defined as a complicated state that related to homeostasis disturbances, over-activity of the sympathetic nervous system and hypothalamus-pituitary-adrenal axis responses. Cardiac preconditioning reduces myocardial damages.

**Objective:**

This study was designed to assess the cardioprotective effects of acute physical stress against ischemia/reperfusion (I/R) injury through the activation of the sympathetic nervous system.

**Methods:**

Thirty-two male Wistar rats were divided into four groups; (1) IR (n = 8): rats underwent I/R, (2) Acute stress (St+IR) (n = 8): physical stress induced 1-hour before I/R, (3) Sympathectomy (Symp+IR) (n = 8): chemical sympathectomy was done 24-hours before I/R and (4) Sympathectomy- physical stress (Symp+St+IR) (n = 8): chemical sympathectomy induced before physical stress and I/R. Chemical sympathectomy was performed using 6-hydroxydopamine (100 mg/kg, sc). Then, the hearts isolated and located in the Langendorff apparatus to induce 30 minutes ischemia followed by 120 minutes reperfusion. The coronary flows, hemodynamic parameters, infarct size, corticosterone level in serum were investigated. P < 0.05 demonstrated significance.

**Results:**

Physical stress prior to I/R could improve left ventricular developed pressure (LVDP) and rate product pressure (RPP) of the heart respectively, (63 ± 2 versus 42 ± 1.2, p < 0.05, 70 ± 2 versus 43 ± 2.6, p < 0.05) and reduces infarct size (22.16 ± 1.3 versus 32 ± 1.4, p < 0.05) when compared with the I/R alone. Chemical sympathectomy before physical stress eliminated the protective effect of physical stress on I/R-induced cardiac damages (RPP: 21 ± 6.6 versus 63 ± 2, p < 0.01) (LVDP: 38 ± 4.5 versus 43 ± 2.6, p < 0.01) (infarct size: 35 ± 3.1 versus 22.16 ± 1.3, p < 0.01).

**Conclusion:**

Findings indicate that acute physical stress can act as a preconditional stimulator and probably, the presence of sympathetic nervous system is necessary.

## Introduction

Ischemic heart disease is the major health problem in the world.^1^ Although reperfusion, which refers to the rapid reestablishment of blood flow, can be one of the most effective methods against lethal injuries,^2^ it is associated with additional myocardial damage.^3^ Many methods have been proposed to diminish the deleterious effect of ischemia/reperfusion (I/R) injuries and increase cardiac endurance. Based on these advances, induction the short-term episodes of I/R or using the pharmacological agents earlier than prolonged I/R period induces cardiac preconditioning which can successfully attenuate cellular necrosis and conserve high levels of energy.^4,5^

Sympathetic nervous system and hypothalamus-pituitary-adrenal (HPA) axis are two coordinated defence systems. They can mediate two-way brain-body communication during stressful situations.^6^ Autonomic system activation contributes to behavioral responses in animals and enables them to regulate homeostasis and improve endurance.^7^ Stress is characterized as a general HPA axis response against potential and deleterious stimuli.^8^ In fact, stress through increasing the activity of HPA axis and corticosterone release plays a critical role in coordinating of neuroendocrine, autonomic and behavioral functions and leads to adaptive responses.^9,10^ The activity of the sympathetic nervous system increases and neurotransmitter secretion alters during the occurrence of stress.^11^ Several body systems such as nervous, cardiovascular and immune systems are influenced by stress. Moreover, significant changes in hemodynamic parameters such as; heart rate (HR) and blood pressure are observed during stress which ultimately may lead to heart diseases.^12^ On the other hand, the release of norepinephrine from the sympathetic nervous system is increased during lethal ischemia and it has a role for inducing of I/R injuries through the generation of hydroxyl free radicals. This current study was designed to evaluate the role of the sympathetic nervous system in mediating acute stress-induced cardioprotection against I/R injury in isolated rat heart.

## Methods

A total of 32 male Wistar rats (200-250g) were kept in an air-conditioned room on a 12 hours light-dark cycle, at 22 ± 2˚C, with free access to water and food. The experimental protocols followed in this study conformed to the Guidelines for the Care and Use of Laboratory Animals published by the National Institutes of Health (NIH Publication No. 85-23, revised 1996) and were further approved by the institutional ethical committee of Tehran University of Medical Sciences (Tehran, Iran).

Stress box apparatus was used for physical stress exposure. It contained stainless bars at the bottom, connected to electroshock device using a connecting cable. Physical stress was induced using electrical foot shock (1mA) for 10 seconds with 50 seconds intervals for one hour. After that, animals were anaesthetized with sodium thiopental (60 mg/kg, i.p),^13^ put on a surgical board. The chest was opened and the surgical silk suture (6-0) placed under the root of the left anterior descending coronary artery (LAD). Finally, the heart was removed from the chest and connected to Langendorff-perfusion apparatus. Heart was perfused in a retrograde manner using Krebs-Henseleit bicarbonate buffer (in mmol/l): sodium bicarbonate = 25, sodium chloride = 118.5, potassium chloride = 4.7, magnesium sulfate = 1.2, glucose = 11, gassed with 95% O_2_ and 5% CO_2_ (pH = 7.3-7.4 at 37˚C). Thereafter, the ends of the suture were passed through a plastic tube to create a snare for ischemia induction. Reperfusion was performed by releasing the snare. Latex fluid- filled balloon was inserted inside the left ventricle and connected to a pressure transducer (Harvard, March-Hugsteten, Germany), the biolab apparatus was used for recording the ventricular pressures. During the surgical procedure, recording was done during three designated periods: 20-30 minutes of the baseline (a period without any manipulation), 30 minutes of the local ischemia and 120 minutes of the reperfusion. After reperfusion, LAD was occluded again; Evans Blue dye (3 mL of 1.5% solution) was administrated to discriminate ischemic zone (the area at risk; [AAR]) from non-ischemic zone.^14^ After freezing (−20°C for 24 hours), heart tissue was sliced into 2mm transverse sections and kept in 1% 2, 3, 5 triphenyltetrazolium chloride (TTC in 0.1 M phosphate buffer, pH = 7.4 Sigma) solution for 15-20 min at 37°C to delineate ischemic from infarct zone.^15^ At the end of the experiments, the ratio of AAR and infarcted size (IS) were calculated by the Photoshop program.

Animals were allotted in 4 groups:

1. IR group (n = 8): Rats were kept in stress box device (without stress exposure) for 1 hour and then, hearts were removed from the chest and subjected to ischemia and reperfusion.
2. Acute stress (St+IR) group (n=8): Rats were exposed electrical feet shock in the stress box for 1 hour and then, hearts were removed from the chest and subjected to ischemia and reperfusion.
3. Sympathectomy (Symp+IR) group (n = 8): chemical sympathectomy was done by injection of a 6-hydroxydopamine (6-OHDA, 100 mg/kg, sc) 24 hours prior to I/R induction.^(16)^
4. Sympathectomy- physical stress (Symp+St+IR) group (n = 8): chemical sympathectomy was done 24 hours prior to physical stress and I/R induction.

We measured serum corticoesterone levels by ELISA method. Moreover, systolic blood pressure was measured via non-invasive technique (Tail Cuff and power lab) to confirm chemical sympathectomy (n = 4).

### Statistical analysis

The sample size and group divisions were defined based on our previous studies.^17^ All data are reported as means ± S.E.M. Normality was checked using Kolmogorov-Smirnov test, SPSS software version 20. One way ANOVA and Tukey post hoc test was done for comparison of parameters between different groups. Analysis of changes in mean values over three times was done using repeated measurement ANOVA within each group. Sample t-test was used to compare systolic blood pressure before and after sympathectomy. Significant changes were considered as p < 0.05.

## Results

### Effect of acute physical stress on coronary flow and heart rate

**Figure 1:**
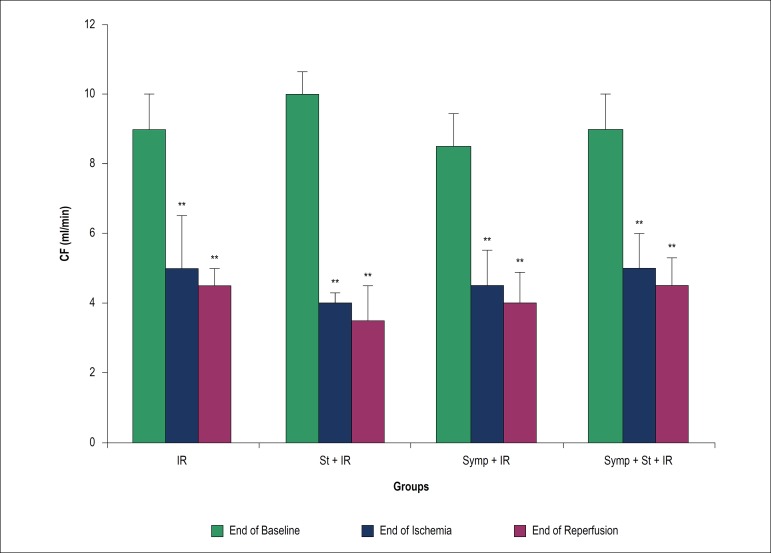
Coronary flow (CF) at the end of baseline, ischemia and reperfusion periods. IR: Ischemia/reperfusion; St: Physical stress; Symp: Symapathectomy. ** p < 0.01 vs baseline phase within the same group.

**Figure 2:**
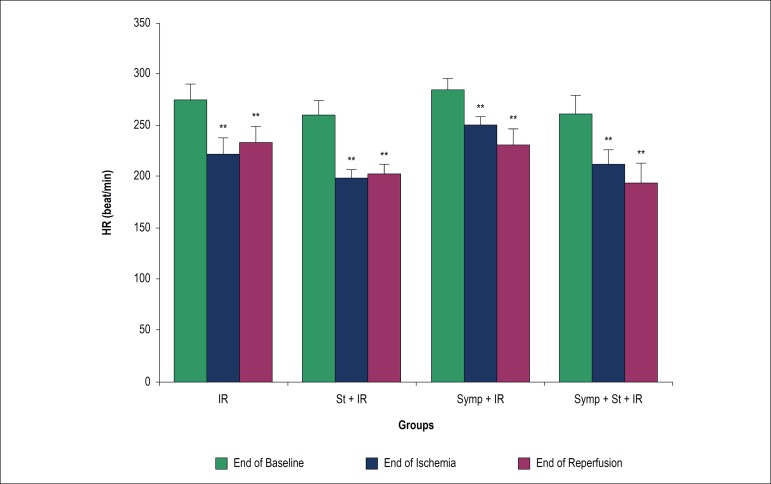
Heart rate (HR) at the end of baseline, ischemia and reperfusion periods. IR: Ischemia/reperfusion; St: Physical stress; Symp: Symapathectomy. ** p < 0.01 vs. baseline phase within the same group.

Figure 1 shows coronary flow (CF) at the end of the baseline, ischemia and reperfusion periods. There are significant differences for CF at the end of ischemia and reperfusion when compared to the end of the baseline period within groups (p < 0.01). HR was significantly decreased at the end of both ischemia and reperfusion in comparison with the end of baseline period within groups (p < 0.01), but no significant change were observed between different groups (Figure 2).

### Effect of acute physical stress on cardiac hemodynamic parameters

**Figure 3:**
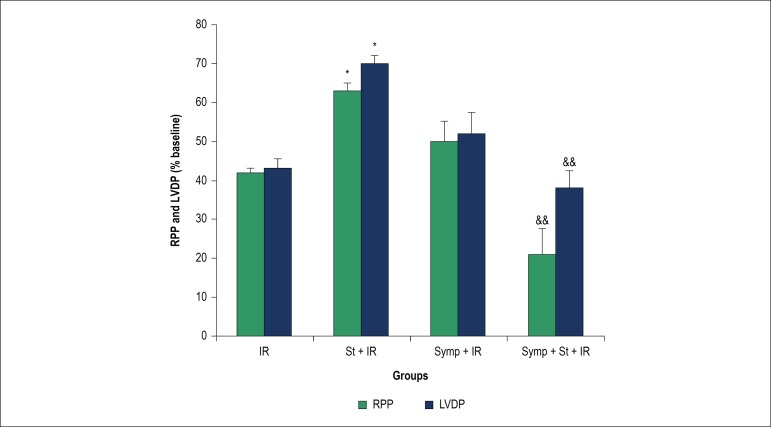
Left Ventricular Developed Pressure (LVDP) and rate product pressure (RPP) at the end of reperfusion period. IR: Ischemia/reperfusion; St: Physical stress; Symp: Symapathectomy. * p < 0.05 compared to IR, && p < 0.01 compared to St + IR.

The left ventricular developed pressure (LVDP, the difference between intraventricular systolic and diastolic pressures), rate product pressure (RPP, LVDP multiplied by HR) and were diminished at the end of reperfusion in comparison to the end of baseline period among groups,

The amount of RPP and LVDP in acute stress group were extremely increased in comparison to IR group (p < 0.05 in induction of chemical sympathectomy before physical stress were considerably decreased RPP and LVDP in comparison to physical stress group p < 0.05, but there is no marked difference between sympathectomy group when compared to IR group (Figure 3).

### Effect of acute physical stress on infarcts size (%IS/AAR)

**Figure 4:**
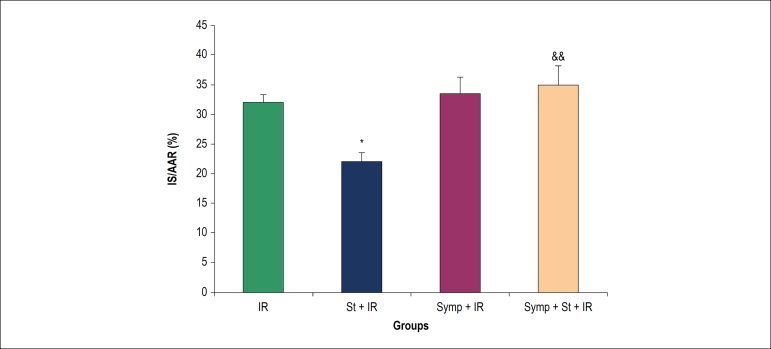
The percentage of infracts size (IS/AAR %). IR: Ischemia/reperfusion; St: Physical stress; Symp: Symapathectomy. * p < 0.05 compared to IR, && p < 0.01 compared to St+IR.

Figures 4 shows the size of the infarct (%IS/AAR) in different groups.

Infarct size was greatly deacreased in acute stress group as compared to the IR group (p < 0.05), but there was no considerable change in chemical sympathectomy group as compared to the control group. Chemical sympathectomy prior to acute physical stress represented no extermly change as compared to IR group, while it has been shown significant reduced infarct size as compared with acute physical stress alone (p < 0.01).

### Effect of acute physical stress on corticosterone level in serum

**Figure 5:**
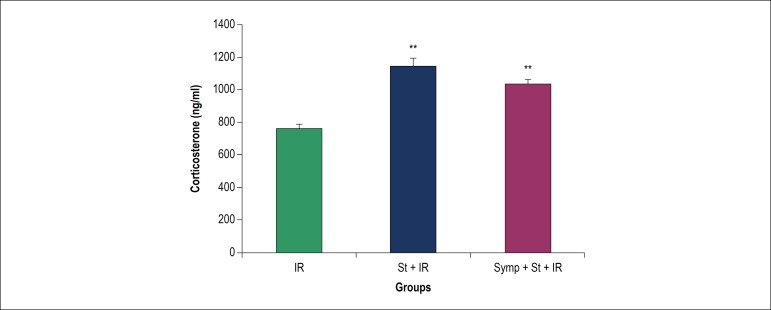
Corticosterone level in serum. IR: Ischemia/reperfusion; St: Physical stress; Symp: Symapathectomy. ** p<0.01 compared to IR.

Figure 5 shows the serum level of corticosterone in different groups. Induction of acute physical stress without or with chemical sympathectomy in St and St+Symp+IR groups could increase the amount of serum corticosterone as compared to the IR group, (p < 0.01).

### Effect of chemical sympathectomy on Systolic blood pressure

**Figure 6:**
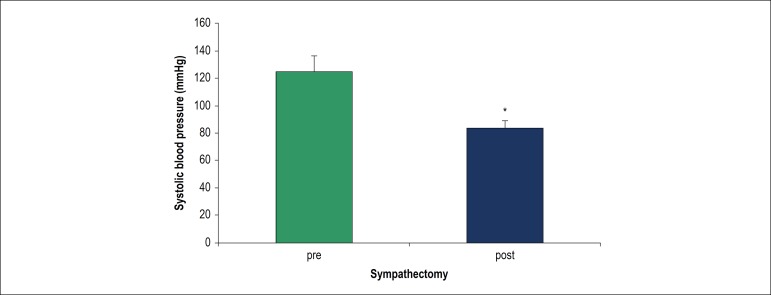
Systolic blood pressure before and after chemical sympathectomy. *p < 0.05 compared to before sympathectomy.

Figure 6 represents the significant reduction of systolic blood pressure after induction of chemical sympathectomy (p < 0.05).

## Discussion

Nowadays daily life is associated with stress that is divided to acute stress and chronic stress, based on exposure duration.^18^ Acute stress mediates several neurogenic pathways.^19^ Electrophysiological recordings revealed that acute stress manifests good effects such as favour heightened arousal and increases cognitive flexibility in an attentional set-shifting task.^20^ In the other view, stress is divided into physical and psychological. A physical stressor such as surgery, trauma and heavy physical activity can trigger many cardiac events.^21^ Psychological stress can affect the cardiovascular system through metabolic, inflammatory and hormonal factors.^22,23^ In this study, we evaluated the effects of acute physical stress prior to sympathectomy on ischemia-reperfusion injuries in isolated rat heart.

### The effects of stress

Our results showed that induction of acute stress prior to ischemia-reperfusion period led to a decrease in the infarct size, improve hemodynamic parameter and increase in the plasma corticosterone level as compared to IR group and Symp+IR group. Two paradoxical theories have been proposed to explain both advantage and disadvantage effect of stress on the heart. Extremely elevated HR, cardiac contractility and peripheral resistant due to exposure to acute stress can increase the cardiac load and oxygen consumption. In contrast, emerging evidences indicate the opposite effect, for example; cold restraint stress induces cardiac cell protection^24^ and it can diminish infarct size as a main parameter of cardiac damage.^25^

In this regard, Abe et al. demonstrated that acute stress attenuates ischemia-reperfusion injury in the kidney through the activation of sympathetic and anti-inflammatory pathway.^26^

Moreover, exposure to intermediate stress involves in cell protection against subsequent lethal ischemia, as a concept, preconditioning phenomenon.^27,28^ It seems that acute physical stress exposure as a preconditioning agent protects the heart against I/R. We observed an increase in RPP and LVDP amounts due to acute stress induction in St+IR group as compared with IR group that indicates acute stress would trigger mechanisms to prepare the body for suitable responses to stimuli because the improvement of the cardiac function is important. Therefore, it seems that the effectiveness of stress induction is associated with; 1. nature of the stressor, 2. stress episode duration, 3. intensity of the stimulus and 4. stress predictability or unpredictability. In fact, each of the above factors effects on the neural and hormonal responses to stress. Our results showed that corticosterone is elevated after stress induction, and in Symp+St+IR group is higher than the IR group. It is well established that stress enhances the activity of the HPA axis which results to increase corticosterone secretion^22,29^ that can be protective as it prepares the organism to deal with challenges. Based on our results sympathectomy did not effect on stress-induced elevated corticosterone possibly because stress affects HPA axis through different mechanisms such as changes in metabolic and inflammatory factors in addition to increased sympathetic nervous system.^22^ Furthermore, this hormone induces changes in immune cells redistribution that enhance immune function.^30^ We showed that infarct size is decreased in St+IR group in comparison to the IR group and the hemodynamic parameters are improved in St+IR group in compare to the IR group. Diminished infarct size led to reducing in cardiac arrhythmia occurrence^31,32^ and also improved cardiac contractility.^33^ It seems the beneficial effects of acute stress induction may relate to the improvement of immune system function due to elevated corticosterone level encounter inflammatory factors, which trigger I/R injuries.

### The effects of sympathectomy

It has been established that exposure to stressful conditions increase autonomic nervous system activity.^30^ The cardioprotection of sympathetic activity has been investigated^34^ and we used chemical sympathectomy after acute stress induction to confirm the protective effects of the sympathetic nervous system. Animals in Symp+IR group were subjected to chemical sympathectomy before induction of I/R and there was no significant change in infarct size in comparison to IR group, indicating that chemical sympathetic denervation has no effect on IR injury. In addition, chemical sympathectomy prior to physical acute stress removed the cardioprotection effect of acute stress on infarct size in Symp+St+IR group that emphasizes the presence of sympathetic system is necessary for cardioprotective effects of acute stress. We found that acute stress induction after chemical sympathectomy could overcome harm effects of deleted sympathetic system on hemodynamic parameters in Symp+St+IR group when compared to Symp+IR group that indicates the essential role of sympathetic system physiological activity in regulating HR, pressure and flow.^35^ Hara and Abiko declared norepinephrine has two opposite effects on ischemia damages according to the duration of ischemia, means that it could protect the heart with short ischemia and increase the injuries with prolonged ischemia.^28^ Positive and negative properties of the sympathetic nervous system are associated with the duration of stimulus exposure. At the long term ischemia, large quantities of norepinephrine are released from the sympathetic nervous system, acting as a source of the free radical and subsequent generation of OH free radical.^36^ Protective effect of norepinephrine can be emerged by producing energy for cardiac muscle in short term ischemia episode.^37^ Moreover, Yohimbine (as an alpha2 receptor antagonist) administration reduced the incidence of arrhythmia through increasing sympathetic norepinephrine release.^37,38^ According to our previous studies, pretreatment with alpha receptor agonist such as phenylephrine could protect the cardiomyocytes against I/R damages in isolated HR.^39^ Activation of protein kinase-C (PKC) signalling pathway^40^ and NO release^41^ by norepinephrine, are involved in the opening of mitochondrial KATP channels, which in turn can reduce mitochondrial calcium load^42^ and will lead to attenuation of norepinephrine beneficial effects. Also, we showed that systolic blood pressure declined after chemical sympathectomy that is compatible with this fact that muscle sympathetic outflow is responsible for the regulation of blood pressure.^43^ The limitations of this study were the method of induction physical stress does not commonly occur in daily life. Unfortunately, the data of corticosterone level in Symp+IR group were missed so we can’t discuss the effect of sympathectomy on corticosterone level and comparison between St+IR group and Symp+St+IR group is not significant. In consistence with our results, Lowrance et al. showed that stress-induced corticosterone level didn’t change following pharmacological sympathectomy.^44^

## Conclusion

The present study showed that induction of physical acute stress before I/R led to cardioprotection and chemical sympathectomy removed this beneficial effect of physical acute stress.
